# Subcellular protein overexpression to develop abiotic stress tolerant plants

**DOI:** 10.3389/fpls.2013.00002

**Published:** 2013-01-21

**Authors:** Mohammad-Zaman Nouri, Setsuko Komatsu

**Affiliations:** ^1^Plant Breeding Department, Rice Research Institute of Iran-Deputy of MazandaranAmol, Iran; ^2^National Institute of Crop Science, National Agriculture and Food Research OrganizationTsukuba, Japan

**Keywords:** subcellular protein, transgenic, overexpression, abiotic stress

## Abstract

Environmental stresses are major factors limiting growth and development of crops. Plants respond to the stresses through a wide range of reactions from morphological changes to alterations in the patterns of protein expression. Understanding the mechanisms involved in the stress response is the first step to develop abiotic stress tolerant crops. Proteomics is a powerful tool in evaluating regulated proteins in the cell under stress and it is an efficient technique in studying stress tolerant plants. Because of the nature of abiotic stress, intracellular compartments play a main role in the stress response. Subcellular proteins such as ion and water transporters, reactive oxygen species (ROS) scavengers, and the proteins related to signaling and transcriptional regulation are frequently reported as being involved in stress tolerance. Overexpression of stress-responsive protein through generation of transgenic plants is one the main practical approaches in production of tolerant plants. In this article, recent studies on transgenic plants overexpressing subcellular proteins are reviewed and the role of organelles and over-expressed proteins is classified.

## Introduction

Adverse environmental conditions threaten normal growth and development of plants. Abiotic stresses mainly including temperature extremes, drought, and salinity detrimentally affect plant growth and crop yield. It was reported that for most crops, abiotic stresses are leading to a reduction in the average yield by more than 50% (Bray et al., 2000). One-third of the world's population resides in water-stressed regions, and because of climate changes, water stress could become more frequent and severe in the future (Manavalan et al., 2009). Adverse abiotic stresses tend to occur together and they are almost never present individually in nature. On the other hand, plant cells likely follow similar mechanisms to cope with abiotic stresses. For instance, cold, drought, and salinity are well-known to the generation of reactive oxygen species (ROS) causing oxidative stress (Dat et al., 2000). Therefore, ROS scavenging genes are candidate in the generation of plants with tolerance to the stress.

At the cellular level, plants adopt a wide range of responses to cope with abiotic stress. The mechanism associated with sensing stress, transduction of stress signals into the cell is well-known, and it represents the initial reaction of plant cells to stress (Desikan et al., 2004). Stress signals are first encountered by the outer parts of the cell under a highly organized process for sensing environmental changes (Heino and Palva, 2003). ROS, which are formed by partial reduction of molecular oxygen during abiotic stress, are recognized as a signal to activate the defense response (Vranová et al., 2002). Transduction of the signal into the cell through cascades alters gene and protein expression, leading to physiological responses. Communication through intracellular compartments plays an important role in this process.

The major subcellular organelles and compartments in plant cells are nucleus, mitochondria, chloroplasts, endoplasmic reticulum (ER), Golgi apparatus, vacuoles, and plasma membrane. The intracellular organelles and their interactions during stressful conditions represent the primary defense response. In fact, communication between organelles and cytosolic and luminal proteins renders the protein composition of organelles dynamic (Agrawal et al., 2011). Most receptor proteins are located in the plasma membrane, and thus the plasma membrane is directly involved in stress sensing (Komatsu, 2008). Intracellular organelles with high capacity for production of ROS, such as mitochondria and chloroplasts are the primary sites for the production of this signaling molecule. ROS can also increase the effects of cellular damage (Dat et al., 2000; Van Breusegem et al., 2001). According to the role of cellular organelles and compartments, there are several key subcellular proteins involved in stress tolerance in plant cells.

Proteome analysis of cellular organelles under abiotic stress indicated that the accumulation of responsible proteins highly improves plant tolerance to the stress. Therefore, overexpression of related genes through the production of transgenic plants is a powerful technique to enhance stress tolerance in plants. Generation of tolerant transgenic plants against abiotic stresses has extensively been discussed in reviews (Wang et al., 2003; Bartels and Sunkar, 2005). However, classification of subcellular-localized proteins and their related genes involved in abiotic stress response is scant. This review highlights the studies in recent decade on abiotic stress tolerant transgenic plants overexpressing subcellular proteins.

## Abiotic stress-related proteins in plant cell

When the plant is exposed to abiotic stress, the proper function of the cell will be highly affected. Cell survival under stress condition directly depends on how the cell can adapt itself to the environment. Cellular defense mechanisms against abiotic stress are mainly controlled by the expression of responsible genes and proteins. Generally, many plants are susceptible to unfavorable environmental conditions. Therefore, overexpression of protective genes might help to reduce the deleterious effects of the stress. Generation of transgenic plants overexpressing the stress-responsive gene and protein will confer stress resistance leading to enhanced plant growth and productivity (Allen et al., 1997). Three major groups of genes are reported to be involved in the stress response. The first group are those that are involved in signaling cascades and in transcriptional regulation. The second group are those having a role in the protection of membranes and proteins and the third group are those involved in water and ion uptake and transport (Wang et al., 2003).

In order to know about major subcellular proteins conferring stress tolerance, it is important to understand the roles of organelles themselves in the cell. Organelles such as chloroplast and mitochondria are mainly responsible for metabolic processes including photosynthesis, photorespiration, oxidative phosphorylation, and the tricarboxylic acid cycle (Taylor et al., 2009). The plasma membrane is mainly involved in stress signal perception, transducing into the cell, and ion and water transport. The tonoplast is involved in ion balance and adjustment of water content. The nucleus has a variety of functions including transcriptional regulation, signaling, and gene regulation (Hossain et al., 2012). Despite the particular roles of organelles, their coordinated functions and interactions are important in the stress response. Hossain et al. (2012) classified the defense-related abiotic stress-responsive proteins into six major groups according to their functions. The protein groups are osmoprotectant regulators, ROS scavengers, ion transporters, water channels, molecular chaperones, and proteolysis-related proteins. Most of the protein groups are functionally attributed to organelles or compartments indicating the importance of subcellular proteins in response to stress conditions.

Apart from changes in the expression of organelle proteins, post-translational modifications (PTMs) of proteins are also known as a defense mechanism against abiotic stress. Redox proteomics is an increasingly emerging branch of proteomics aimed at identifying PTMs, particularly under stressful conditions. Recent proteome studies analyzed a number of redox-targets at organelles, which offered a view of the proteome modifications that are regulated by abiotic stress. For example, several proteins are identified as a target for S-nitrosylation in peroxisomes (Ortega-Galisteo et al., 2012) and mitochondria (Camejo et al., 2012) of pea plants under abiotic stress conditions. The modification of organelle proteins could regulate H_2_O_2_ level or modulate the respiratory and photorespiratory pathways in the plant.

## Overexpressing of organelle proteins in transgenic plant improves tolerance to abiotic stress

**Figure 1:**
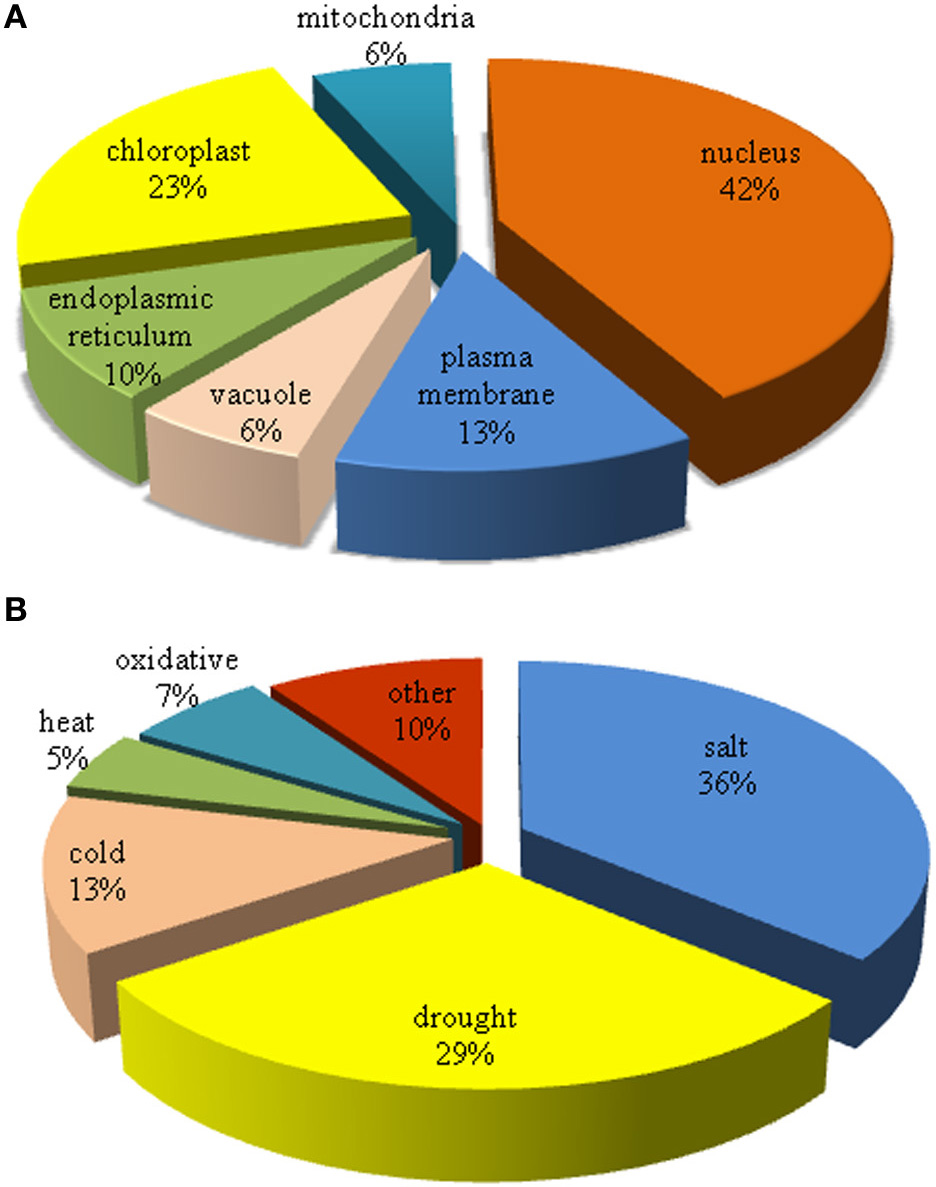
**Subcellular localization of overexpressed protein and type of abiotic stress is classified. (A)** Percentage of organelles in which overexpressed proteins were localized. **(B)** Distribution of abiotic stresses in which transgenic plants were tolerant.

Transgenic plants overexpressing the genes encoded subcellular-localized proteins are classified (Table 1). Stress-responsive genes or proteins were arranged according to the localization of the overexpressed protein in the cell. Nucleus, chloroplast, plasma membrane, ER, mitochondria, and vacuole are the organelles and compartments in which the presence of overexpressed proteins was confirmed (Table 1). Among the organelles, near half (42%) of the overexpressed proteins was localized in the nucleus (Figure 1A). This means that the genes encode nuclear proteins were highly considered in the generation of transgenic plants. According to the role of nucleus protein under abiotic stress, it can be postulated that the molecular mechanism of stress tolerance in most of the studied transgenic plants is based on transcriptional regulation, signaling, and gene regulation. Other overexpressed proteins were mainly localized in chloroplast (23%), plasma membrane (13%), and ER (10%) (Figure 1A). This classification performs a preliminary ranking of the importance of organelles in response to abiotic stress, regardless of the function of overexpressed protein.

**Table 1:** **Overexpression of subcellular proteins in transgenic plants to enhance tolerance to abiotic stress (Since 2000)**.

**No.**	**Localization**	**Protein (gene)**	**Abiotic stress**	**Effects on plant/cellular mechanism**	**Plant species**	**References**
1	Nucleus	Cold shock protein (*CSP*)	Cold, heat, drought	Improve in plant growth, yield	*Arabidopsis thaliana, Oryza sativa*	Castiglioni et al., 2008
		R1R2R3 MYB TF (*OsMYB3R-2*)	Freezing, drought, salt	Tolerance to NaCl and ABA during seed germination	*Arabidopsis thaliana*	Dai et al., 2007
		Late embryogenesis abundant protein (*OsLEA3-2*)	Salt, drought	Improve in germination speed, plant growth	*Oryza sativa*	Duan and Cai, 2012
		R2R3 MYB transcription factor (*AtMYB44*)	Drought, salt, cold	Water loss reduction	*Arabidopsis thaliana*	Jung et al., 2008
		ethylene-responsive factor like protein 1 (*CaERFLP1*)	Salt	Enhance tolerance	*Nicotiana tabacum*	Lee et al., 2004
		TaMYB2A (*TaMYB2A*)	Drought, salt, freezing	Enhance cell membrane stability, photosynthetic rate, reduction of osmotic potential	*Arabidopsis thaliana*	Mao et al., 2011
		OsNAC6 (*OsNAC6*)	Drought, salt	Transcriptional activator	*Oryza sativa*	Nakashima et al., 2007
		WRKY-type transcription factor (*TaWRKY2, TaWRKY19*)	Drought, salt	Regulation of STZ or DREB2A-mediated pathways	*Arabidopsis thaliana*	Niu et al., 2012
		Ethylene-responsive element binding protein (*AtEBP*)	Oxidative, heat	Suppression of Bax-induced cell death	*Nicotiana tabacum*	Ogawa et al., 2005
		Trihelix transcription factor (*GmGT-2A, GmGT-2B*)	Salt, drought, freezing	Improve in seedling morphogenesis	*Glycin max*	Xie et al., 2009
		R2R3-MYB transcription factor (*OsMYB2*)	Drought, salt, cold	Accumulation of more soluble sugars and proline	*Oryza sativa*	Yang et al., 2012
		Basic leucine zipper transcription factor (*AtbZIP24*)	Salt	Osmotic and ionic balance	*Arabidopsis thaliana*	Yang et al., 2009
2	Chloroplast	β-carotene hydroxylase (*DSM2*)	Drought	Control of the xanthophyll cycle and ABA synthesis	*Oryza sativa*	Du et al., 2010
		Glycine betaine (*codA*)	Salt, cold, oxidative	Enhance in plant growth, repair of photo-damaged PSII	*Oryza sativa, Lycopersicon esculentum*	Su et al., 2006; Park et al., 2007
		Homogentisate phytyltransferase (*HPT1*)	Light	Tocopherol regulation	*Arabidopsis thaliana*	Collakova and DellaPenna, 2003
		Betaine aldehyde dehydrogenase (*BADH*)	Salt, oxidative, cold	Higher photosynthetic activity, reduction of ROS	*Ipomoea batatas*	Fan et al., 2012
		Plastidal protein synthesis elongation factor (*Zmeftu1*)	Heat	Reduced thermal aggregation of leaf proteins, reduced heat injury to thylakoids, enhanced rate of CO_2_ fixation	*Triticum aestivum*	Fu et al., 2008
		Chloroplast small heat shock protein (*Cpshsp*)	Cold	Less electrolyte leakage and less destruction of chlorophyll, higher photosynthetic rate	*Lycopersicon esculentum*	Wang et al., 2005
		Aldehyde dehydrogenase (*ALDH3I1*)	Salt, drought	ROS scavenger, reduced the level of lipid peroxidation	*Arabidopsis thaliana*	Kotchoni et al., 2006
3	Plasma membrane	Na^+^/H^+^ antiporter (*SOS1*)	Salt	Less Na^+^ accumulation, improve yield	*Arabidopsis thaliana, Zea maiz*	Shi et al., 2002; Chen et al., 2007
		Aquaporin (*PIP1b*)	Drought (-)^*^	Higher plant growth, transpiration rate, stomatal density and photosynthetic efficiency, plant vigore	*Nicotiana tabacum*	Aharon et al., 2003
		Nodulin 26-like intrinsic protein (*TaNIP*)	Salt	Higher K^+^, Ca^2+^ and proline contents and lower Na^+^ level	*Arabidopsis thaliana*	Gao et al., 2010
		Sucrose non-fermenting 1-related protein kinase 2 (*TaSnRK2.8*) ^**^	Drought, salt, cold	Higher relative water content, cell membrane stability, PSII activity	*Arabidopsis thaliana*	Zhang et al., 2010
4	Endoplasmic reticulum	E3 ubiquitin ligase (*Rma1H1*)	Drought	Inhibiting aquaporin trafficking	*Arabidopsis thaliana*	Lee et al., 2009
		Annexin (*AnnAt1 and AnnAt4*)	Drought, salt	Improve in plant growth	*Arabidopsis thaliana*	Huh et al., 2010
		BiP (soy *BiP*)	Oxidative	Tolerance to the glycosylation inhibitor tunicamycin, tolerance to water deficit	*Nicotiana tabacum*	Alvim et al., 2001
5	Mitochondria	Glycine-rich RNA-binding protein 2 (*GRP2*)	Salt, cold, osmotic stress	Higher seed germination, seedling growth and freezing tolerance	*Arabidopsis thaliana*	Kim et al., 2007
		Uncoupling protein (*AtUCP1*)	Drought, salt	Higher germination, photosynthesis, respiration, leaf water content	*Nicotiana tabacum*	Begcy et al., 2011
6	Vacuole	Na^+^/H^+^ antiporter (*AtNHX1, TNHX1*)	Salt, drought	Higher photosynthesis, nitrogen assimilation	*Arabidopsis thaliana, Gossypium hirsutum*	Brini et al., 2007; He et al., 2005
		ERD six-like1 (*ESL1*)	Drought, salt, ABA	Efflux of hexoses from the vacuole	*Arabidopsis thaliana*	Yamada et al., 2010

* Minus indicates the negative effects of overexpressed protein under stress condition.

** This protein co-localized in nucleus, cytoplasm, and plasma membrane.

Classification of the transgenic plant species indicated that out of 30 overexpressed proteins, *Arabidopsis*, rice, and tobacco were applied in 16, 6, and 5 studies, respectively (Table 1). Interestingly, while overexpressed proteins of *Arabidopsis* and tobacco were localized in diverse organelles, the overexpressed proteins of rice were only localized in the nucleus and chloroplast. Bartels and Sunkar (2005) classified stress-responsive genes contributing to drought or salt tolerance in transgenic plants. They reported a similar order for a number of successful transgenic *Arabidopsis*, tobacco, and rice. This result indicates that despite great advances in the generation of transgenic plants, most of the transgenic plants are among the model plants.

Overexpression of a specific protein in transgenic plant confers a level of tolerance to the plant. A diverse range of stresses that are mainly related to unfavorable environmental conditions is classified as abiotic stress. Overexpressed gene or protein in most of the transgenic plants is contributing to salt (36%) and drought (29%) tolerance which means 65% of all studies were only focused on salt and drought tolerance. Tolerance to other abiotic stresses such as cold (13%), oxidative (7%), and heat (5%) were relatively less reported (Figure 1B). Manavalan et al. (2009) described the importance of water stress at the present and in the future. Therefore, researchers are working more on the generation of salt and drought stress tolerant transgenic plants.

## Effects of protein overexpression on cellular mechanisms of plant

A wide range of responses has been reported in transgenic plants subjected to abiotic stress. Alterations in morphology, physiology, and cellular mechanisms of transgenic plants are summarized (Table 1). The articles listed in the table, mainly did not comprehensively characterize cellular responses to the stresses. Therefore, it is difficult to perform functional classification of the overexpressed genes or proteins. However, growth enhancement in transgenic plants compared to wild type can be considered as a general morphological response. Improvement of the photosynthetic activity is another mechanism involved in stress tolerance. Proteins including MYB transcription factor (TaMYB2A) (Mao et al., 2011), glycine betaine (Park et al., 2007), betaine aldehyde dehydrogenase (Fan et al., 2012), chloroplast small heat shock protein (Wang et al., 2005), Sucrose non-fermenting 1-related protein kinase 2 (Zhang et al., 2010), and even Na^+^/H^+^ antiporter (Brini et al., 2007) enhance photosynthetic activity. The proteins related to photosynthesis were not only localized in the chloroplast but also they were overexpressed in the other organelles.

Overexpression of some proteins in transgenic plant might have negative effects on plant tolerance to abiotic stress. Creissen et al. (1999) reported that in transgenic tobacco overexpressing chloroplast-targeted γ-glutamylcysteine synthetase (γ-*ECS*), foliar levels of glutathione were significantly raised. This protein as a major antioxidant in most aerobic organisms supposed to protect the photosynthetic apparatus from oxidative damage. Paradoxically, however, increased glutathione biosynthetic capacity in the chloroplast resulted in greatly enhanced oxidative stress possibly by failure of the chloroplast homeostatic mechanism (Creissen et al., 1999). Transgenic tobacco overexpressing aquaporin (*PIP1 b*) is another example of the susceptibility of transgenic plant to abiotic stress. Aquaporin improves plant growth rate, transpiration rate, stomatal density, and photosynthetic efficiency under favorable growth conditions. However, overexpression of aquaporin reduced plant growth under drought stress because of rapid wilting (Aharon et al., 2003). Therefore, two main strategies in the generation of successful transgenic plants are; (1) consideration of the cellular and subcellular interactions of overexpressed protein; (2) selection of stress-specific target gene or protein according to the main role of the protein under stress condition.

## Conclusions and future persective

Abiotic stress is one of the main challenges in expansion of planting area worldwide. Application of omics technology such as proteomics, transcriptomics, and metabolomics together with bioinformatics are frequently reported in plant abiotic stress studies (Moumeni et al., 2011; Hakeem et al., 2012). Proteomics improved the efficiency of conventional breeding by identification of the stress-responsive proteins. Overexpression of specific stress-responsive protein through the generation of transgenic plant is an efficient technique which has been successfully applied in model plants. In this review, a survey of recent decade studies (from 2000) to generate transgenic plants overexpressing subcellular-localized proteins is presented. Classification of transgenic plants according to the organelle indicated that 42% of the overexpressed proteins were localized in the nucleus. Therefore, the nucleus functions such as transcriptional regulation, signaling, and gene regulation are more considered in the generation of the plants with tolerance to abiotic stress.

Furthermore, 23 out of the 30 stress-responsive proteins were overexpressed only in *Arabidopsis*, rice, or tobacco. This result suggests that despite great advances in the generation of transgenic plants, this technique is mainly applied in the model plants. Therefore, one of the challenges in the future would be adopting the technology of protein overexpression in economically relevant crops. However, our knowledge about cellular mechanisms against abiotic stress as a prerequisite needs to be improved. Rapidly developing omics technology has the potential to decipher the functions of cell and intracellular organelles against abiotic stress in future studies.

### Conflict of interest statement

The authors declare that the research was conducted in the absence of any commercial or financial relationships that could be construed as a potential conflict of interest.
